# Buprenorphine deaths confirmed by toxicology reveal a low proportion of opioid agonist treatment before death in Finland

**DOI:** 10.1007/s00414-024-03273-5

**Published:** 2024-06-24

**Authors:** Claudia Mariottini, Margareeta Häkkinen, Pirkko Kriikku, Ilkka Ojanperä

**Affiliations:** 1 Department of Forensic Medicine, University of Helsinki, P.O. Box 21(Haartmaninkatu 3), Helsinki, 00014 Finland; 2 Forensic Toxicology Unit, Finnish Institute for Health and Welfare, P.O. Box 30, Helsinki, 00271 Finland; 3 A-Clinic Ltd, Kuortaneenkatu 2, Helsinki, 00510 Finland

**Keywords:** Buprenorphine-related death, Concomitant drugs, Opioid agonist treatment, Poisoning, Poly-drug misuse, Youth

## Abstract

We studied opioid agonist treatment (OAT) status before buprenorphine-related death in Finland, where buprenorphine is the principal OAT medicine and also the most misused opioid, through a retrospective population-based study using medico-legal cause-of-death investigation and OAT patient records. The study included all death cases (*N* = 570) between 2018 and 2020 with a buprenorphine or norbuprenorphine finding in post-mortem toxicology and with known drug misuse history or concomitant findings of illicit drugs. Of the deceased, 10% had received OAT in the year before death. Less than 1% of individuals < 25 years had received OAT, whereas the proportion in individuals ≥ 25 years was 13% (*p* < 0.001). There were significantly more females and more fatal poisonings (*p* < 0.001) among those < 25 years than among those ≥ 25 years. OAT medication at the time of death was sublingual buprenorphine-naloxone in 74% and subcutaneous buprenorphine in 23%. Except for significantly fewer benzodiazepine findings among those receiving OAT, minimal differences were found in terms of age, gender, cause and manner of death, or concomitant substance use between the deceased in and outside of OAT. Concomitant misuse of benzodiazepines, psychostimulants, alcohol, and gabapentinoids was frequent both in and outside of OAT and likely contributed to the death. These results suggest that access to OAT especially for young people and treatment of multiple addictions should be improved. Comprehensive information from medico-legal cause-of-death investigation as a starting point, combined with subsequent ante-mortem patient records, proved to be a successful approach to shed light on the Finnish scene of buprenorphine mortality.

## Introduction

Opioid use disorder (OUD) is a chronic relapsing disorder associated with significantly increased rates of morbidity and mortality. The individual and public health disease burdens of OUD are substantial. Globally, 26.8 million people were estimated to be living with OUD in 2016, with more than 100,000 opioid overdose deaths annually [1].

Opioid agonist treatment (OAT) is an effective treatment for OUD, which aims to stabilize the lives of people with this condition and reduce the harm related to their drug use. OAT is associated with lower rates of overdose death and all-cause mortality [2–5]. The treatment is usually conducted with the opioid agonists buprenorphine (BUP) or methadone (MET).

BUP is gaining interest worldwide because of its increasing use in OAT. The use of BUP is characterized by a ceiling effect with respect to respiratory depression and is thus associated with lower rates of mortality than MET [6]. A study of the relative safety of BUP and MET for OAT conducted by Marteau et al. [7] reported that BUP was six times safer than MET regarding the risk of overdose in the general population. However, despite its relative safety, in several countries BUP has been associated with misuse, diversion and poisoning deaths since it became more widely available in OAT during the mid-1990s [8, 9]. In the United States and many other countries, increasing concerns have been raised about the disadvantages associated with BUP therapy [10]. Accidental and intentional BUP toxicity deaths occur among both OAT patients and opioid users outside of treatment, with intravenous injection and concomitant sedative drug use being recognized as risk factors [11, 12].

In Finland, BUP is a more commonly prescribed OAT medicine than MET, and also the most misused opioid in this country [13–17]. For the past two decades, it has been the main opioid causing fatal poisonings, with an annual mortality rate of 1.9–2.7/100.000 in the general population aged between 15 and 64 during 2010–2014 [18]. Most Finnish opioid users started and have continued their opioid misuse with BUP. The early introduction of BUP in the 1990s by two general practitioners with questionable OAT methods likely influenced the evolution of the illicit drug scene towards frequent parenteral misuse of BUP and much less of that of heroin, which instead, since 2005, has been causing only 1–2 deaths annually [18]. The main misused BUP products are the illicit pharmaceutical-grade mono-BUP tablets that are smuggled into the country from abroad, especially from France [12, 18]. The parenteral use of the combination product with naloxone (BUP-NAL) is less frequent, but it may as well be subject to misuse and cause fatal poisonings [10, 18]. Subcutaneous implants and subcutaneous extended-release injections are expected to have lower misuse potential. The use of the weekly or monthly injectable BUP started in Finland in January 2019. At the end of 2019, injectable BUP covered 18.9% of all OAT with BUP [19]. Since then, the proportion of injectable BUP has increased markedly, but exact numbers are not available.

Given that BUP is both the most administered OAT medication and the most misused opioid, we aimed to investigate the differences in BUP-related deaths during and outside of OAT in Finland. In the absence of a unified register for OAT, we sought to obtain for the first time this information by contacting substance misuse service units nationwide for patient records.

The specific aims of the study were

- To investigate how many of the deceased were receiving OAT;
- To compare those individuals who were in OAT to those who were not, in terms of sex and age, OAT and other prescribed medication, type of OAT, post-mortem findings and use of non-prescribed drugs;
- To provide evidence-based information about the BUP epidemic.

## Methods

### Medico-legal cause-of-death investigation

The research material available for this study included comprehensive toxicological laboratory analysis results and the following information extracted from the death certificate issued by the forensic pathologist: time of death, age, sex, cause of death, manner of death, main autopsy findings and a brief description of the circumstances of death. A general description of the toxicological panel used has been presented previously [20]. The Finnish Institute for Health and Welfare (THL) maintains the national post-mortem toxicology database, in which all the above results are collected.

The forensic pathologist’s diagnosis of BUP poisoning as the underlying cause of death was based on all available case information in addition to toxicology. At autopsy, froth in the airways and pulmonary oedema were typical pathological findings. The median peripheral blood BUP concentration in fatal BUP poisonings was 4 ng/mL, which is somewhat higher than the previously published median fatal concentrations of the order of 1–2 ng/mL [21–24]. Reference therapeutic concentrations for BUP in whole blood or plasma have been reported to range widely from 1 to 20 ng/mL, while BUP fatalities have similar reported blood concentrations from 1.1 to 29 ng/mL [21]. Since the BUP concentration ranges overlap in post-mortem blood between poisonings and other causes of death, diagnosing BUP poisonings was in difficult cases supported by the norbuprenorphine to BUP concentration ratio in blood and urine. The urinary ratio has previously been shown to be below one in most BUP poisonings and above one in most deaths with other causes of death [22–24].

### Data collection

In this population-based retrospective study, we investigated all deaths (*N* = 604) in the THL national post-mortem toxicology database between 2018 and 2020 with a BUP or norbuprenorphine finding and a drug misuse history, based on background information, autopsy findings or concomitant findings of illicit drugs. These deaths, regardless of the cause and manner of death and without BUP being necessarily implicated in the cause of death, are in this study referred to as BUP-related deaths.

We sent an inquiry questioning whether the deceased had received OAT during 2017–2020 to 114 Finnish substance misuse service units offering OAT, according to the last municipality of residence of the deceased. The unique personal identification number assigned to all Finnish citizens at birth and given to permanent residents in Finland made linking the deceased persons to their respective patient records feasible. If in treatment, we asked to provide patient records containing information on the following: treatment plan of the OAT, OAT duration, OAT medication and prescribed supportive medication, and concomitant misuse of other non-prescribed and illicit drugs.

In total, we received the requested information for 570 individuals (94%) that we divided into the following three groups:

Group A: Individuals who were receiving OAT at the time of death;

Group B: Individuals who had received OAT less than 12 months before death, but not at the time of death;

Group C: Individuals who had not received OAT or had received OAT more than 12 months before death.

We considered OAT as terminated, when the treatment unit had recorded the treatment as ceased in the patient’s record.

In this study, we refer to individuals under 25 years of age as young people, following the age classification used by the European Monitoring Centre for Drugs and Drug Addiction in the European Drug Report [25].

### Statistical analysis

We performed all statistical analyses using the Statistical Package for the Social Sciences (SPSS version 28.0, IBM, USA). Since the values within a group were generally not normally distributed, we used medians and ranges to characterize the data. We used cross tabulation, the Kruskall-Wallis test and the Mann-Whitney test to test for group differences, and the Bonferroni correction to correct for multiple comparisons. We considered a p-value of < 0.05 statistically significant for all analyses.

### Ethical issues

We carried out the study on the basis of the research permit THL/1084/0.01.00/2020, issued by the Finnish Institute for Health and Welfare, Finland.

## Results

### Treatment status

As a result of our inquiry, we received information on 574 out of 604 individuals (95% of total cases) from the substance misuse service units. Of these, 514 individuals (90%) had not received OAT within 12 months before death (Group C). Of the remaining 60 individuals (10%), 43 (8%) were receiving OAT at the time of death (Group A) and 13 (2%) had been in OAT less than a year before death but not at the time of death (Group B). We excluded from the study the remaining four (1%) individuals, who had been in OAT, because no other information was available on them, resulting in the final number of 570. The number of individuals included in the study was similar in each of the studied three years (*p* > 0.05).

In Group B, OAT was ceased against the patient’s will in 3 cases, whereas 9 patients ceased OAT themselves. For one patient, the procedure of OAT cessation was unknown. Only one individual died within one month after OAT cessation. The mean and median time between OAT cessation and death in Group B was 3.5 and 2.5 months, respectively. All of the Group B patients had experienced difficulties in committing to OAT, and none had been engaged in a planned, slow medication taper.

### Characteristics of decedents

Of all the individuals studied, 83% (*N* = 472) were male. The proportion of females was 19%, 15% and 17% in groups A, B and C, respectively. The proportion of females did not vary significantly between the groups or between the studied years.

**Fig. 1:**
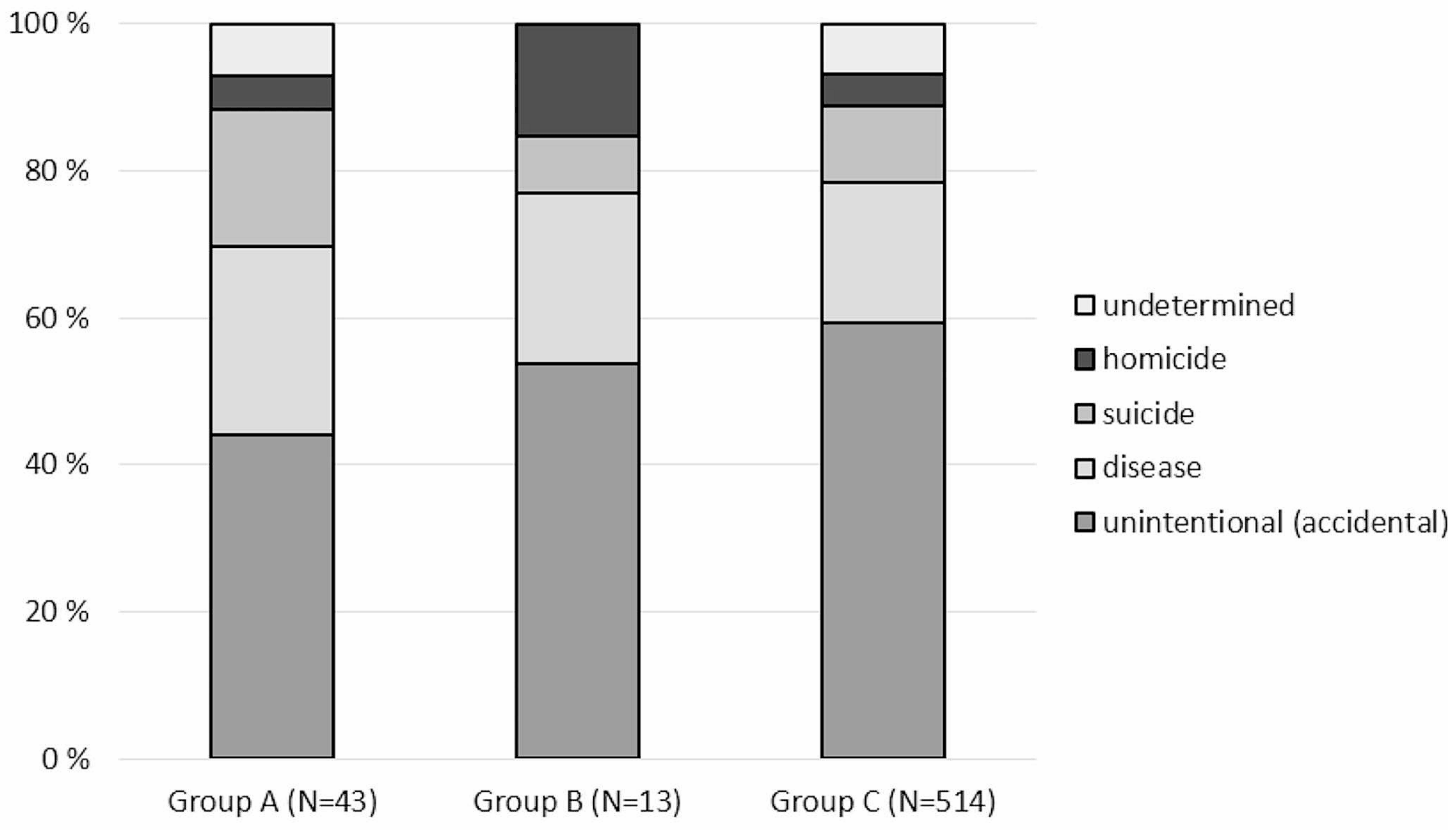
Manners of death in groups **A**–**C**. Group **A**: individuals who were receiving opioid agonist treatment at the time of death; Group **B**: individuals who had received opioid agonist treatment less than one year before death; Group **C**: individuals who had not received opioid agonist treatment or had received opioid agonist treatment more than one year before death. In Group **C**, undetermined part also includes one case with another manner of death

The proportions of different manners of death in each group are shown in Fig. 1. The proportion of poisonings was highest in Group C, but the difference was not statistically significant, as was the case with all manners of death (Table 1). Non-poisoning deaths consisted mainly of deaths due to natural causes.

**Table 1:** Proportion of buprenorphine poisonings in groups A–C

Cause of death	Group A		Group B		Group C		All
	*N*	(%)	*N*	(%)	*N*	(%)	*N*
Buprenorphine poisonings	23	53	7	54	274	53	304
Other poisonings	0	0	0	0	40	8	40
No poisoning	20	47	6	46	200	39	226
**All**	43	100	13	100	514	100	570

Group A: individuals who were receiving opioid agonist treatment at the time of death; Group B: individuals who had received opioid agonist treatment less than one year before death; Group C: individuals who had not received opioid agonist treatment or had received OAT more than one year before death

**Fig. 2:**
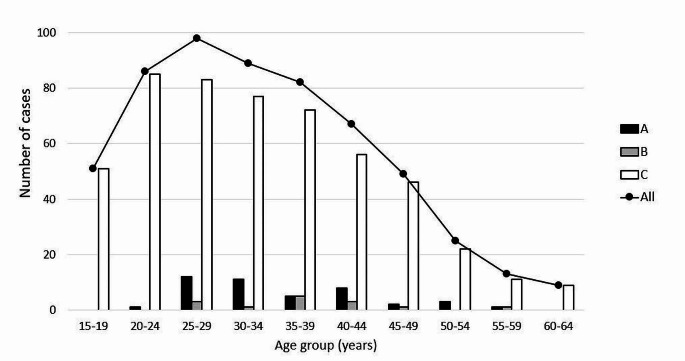
Age distribution of buprenorphine user deaths in groups **A**–**C**. Group **A**: individuals who were receiving opioid agonist treatment at the time of death; Group **B**: individuals who had received opioid agonist treatment less than one year before death; Group **C**: individuals who had not received opioid agonist treatment or had received OAT more than one year before death

Figure 2 illustrates the number of BUP user deaths divided into 5-year age groups. The age of two deceased persons belonging to Group C was not known. Among all studied individuals, the median age was 32 years, and the median age of females (28.5 years) was significantly lower than the median age of males (33.0 years) (*p* < 0.001).

Of all studied individuals, 137 (24%) were under 25 years. The proportions of those under 25 years in each group were the following: Group A – 2.3% (*N* = 1), Group B – 0% and Group C – 26% (*N* = 136). There were significantly more fatal poisonings among those under < 25 years than among those ≥ 25 years (*p* < 0.001). Less than 1% of those < 25 years were or had been in OAT, whereas among those ≥ 25 years, the proportion was 13% (*p* < 0.001). There were significantly more females among those < 25 years (26%) than among those ≥ 25 years (17%) (*p* = 0.001). All 15 individuals under 18 years belong to Group C.

Among the individuals in Group A, 16 (37%) were in a rehabilitating OAT program; 8 (19%) were in a harm-reducing OAT program; and in 19 (44%) cases, the type of OAT was not mentioned or not defined. In Group B, the proportions of the different OAT programs were, respectively, 2 (15%), 5 (28%) and 6 (46%).

As shown in Table 1, in approximately half of the cases in each group, drug poisoning with BUP was implicated as the main cause or one of the main causes of death. The prevalence of fatal poisoning was similar in all groups, and no significant differences were found in the causes of death.

### OAT medication and concomitant substance use

In Group A, the last OAT medication used before death was the sublingual combination product BUP-NAL (Suboxone® or Bunalict®) in 32 (74%) cases, subcutaneous BUP injection (Buvidal®) in 10 (23%) cases and oral MET in 1 (2%) case. In Group B, the last OAT medication was sublingual BUP-NAL in 8 (62%) cases and oral MET in 5 (38%) cases.

The concomitant drugs revealed by post-mortem toxicology in each group are shown in Table 2. A comparison between drug prescriptions and post-mortem toxicology findings showed 32 (74%) cases in Group A with findings of benzodiazepines and/or gabapentinoids and/or opioids not prescribed. When also taking into consideration psychostimulants, cannabinoids, other illicit drugs and alcohol, 38 (88%) and 13 (100%) cases in Group A and Group B, respectively, showed findings of non-prescribed substances. There were significantly fewer benzodiazepine findings in Group A than in the other groups (*p* < 0.05). No other significant differences were found between the groups in the prevalence of post-mortem findings of other pharmacological drug groups or alcohol. We investigated findings of non-prescribed drugs in groups A and B by comparing post-mortem findings to each patient’s list of prescribed drugs (Table 3).

**Table 2:** Post-mortem toxicological findings in groups A–C

Substance group	Group A (*N* = 43)		Group B (*N* = 13)		Group C (*N* = 514)	
	*N*	(%)	*N*	(%)	*N*	(%)
Alcohol	15	35	3	23	206	40
Benzodiazepine(s)	36	84	13	100	471	92
Gabapentinoid(s)	14	33	5	38	233	45
Opioid(s)*	6	14	4	31	5	1
Psychostimulant(s)	24	56	9	69	259	50
Cannabinoid(s)	12	28	4	31	181	35
Other illicit drug(s)**	2	5	2	15	9	2

* Other than buprenorphine

** Includes findings of gamma hydroxybutyrate (GHB), lysergic acid diethylamide (LSD), 2-fluorodeschloroketamine, and/or 3-methoxyphencyclidine (3-MeO-PCP)

Group A: individuals who were receiving opioid agonist treatment at the time of death; Group B: individuals who had received opioid agonist treatment less than one year before death; Group C: individuals who had not received opioid agonist treatment or had received opioid agonist treatment more than one year before death

**Table 3:** Findings of non-prescribed drugs in groups A and B

Substance group	Group A		Group B	
	*N*	(%)	*N*	(%)
**Hypnosedative(s)**	**31**	**72**	**12**	**92**
Alprazolam	12	28	9	69
Chlordiazepoxide	0	0	1	8
Clobazam	0	0	1	8
Clonazepam	15	35	6	46
Diazepam	21	49	4	31
Lorazepam	4	9	0	0
Nitrazepam	1	2	0	0
Oxazepam	3	7	1	8
Temazepam	2	5	1	8
Zopiclone	1	2	0	0
**Gabapentinoid(s)**	**11**	**26**	**4**	**31**
Gabapentin	4	9	2	15
Pregabalin	9	21	3	23
Opioid(s)*	**5**	**12**	**13**	**100**
Buprenorphine	1	2	13	100
Heroin	1	2	0	0
Methadone	2	5	1	8
Morphine	1	2	0	0
Oxycodone	1	2	3	23
Tramadol	1	2	1	8

* Opioid findings other than prescribed opioid agonist treatment medication (buprenorphine or methadone)

Multiple findings of non-prescribed drugs may exist in each case. Prescribed drugs were excluded from table for reasons of clarity. Group A: individuals who were receiving opioid agonist treatment at the time of death; Group B: individuals who had received opioid agonist treatment less than one year before death; Group C: individuals who had not received opioid agonist treatment or had received opioid agonist treatment more than one year before death

## Discussion

We investigated for the first time the role of OAT before BUP-related death in a population-based study in Finland, a country where BUP is both the principal OAT medicine and the opioid most often involved in drug-related deaths. Only 8% of the deceased had been in OAT at the time of death. For those under 25 years, the proportion was less than 1%. Previous studies have shown that mortality is highest within 2–4 weeks after OAT cessation [3, 4]. In our study, however, only 1 out of 13 patients died within a month after OAT cessation.

According to the European Drug Report [25], in 2021, Finland had the highest proportion of young individuals among those who died of fatal drug poisoning when compared to other EU countries. Although comparisons between countries are problematic for various reasons, such as differences in the coverage of cause-of-death investigations, the increasing trend in drug-related deaths among adolescents is alarming. Our study reflects this phenomenon in a very low number of people under 25 years that had received OAT before death. The inability to recognize symptoms related to overdose and a general perception of immunity to harm may add to the vulnerability of young people to fatal overdose [26]. Significantly more females than males died under the age of 25 years. This interesting finding may be connected to earlier observations in studies that have shown young females being at greater risk for prescription drug misuse [27, 28]. The underlying factors that have been suggested to expose young females to more serious substance abuse problems than males are likely to be similar in our study, consisting of more extensive problems in several aspects of life [27].

Based on this and previous studies, the Finnish problem of frequent fatal BUP poisonings is exceptional. In Finland, BUP-related deaths are mainly not associated with the diverted OAT drugs, but the illicit market consists of mono-BUP tablets smuggled into the country from abroad [18]. Bech et al. [29] reported that deceased OAT patients prescribed BUP in Norway tended to replace their agonist with full agonists. We did not find such a phenomenon, which further emphasizes the supremacy of mono-BUP on the Finnish drug scene. In other countries with an increasing number of patients in OAT with BUP, deaths related to BUP have remained at a lower level [30] and involved older individuals [11]. Most international studies emphasize the safety of BUP compared to MET. According to these studies, non-prescribed use of BUP-NAL is rare and focuses on managing withdrawal symptoms rather than seeking euphoria [31–33].

A comparison with Sweden gives an idea of the differences in OAT between these neighbouring countries with similar social systems. According to a Swedish report on poisonings by illegal and prescription drugs, the proportion of individuals that had received OAT less than five years before an unintentional poisoning death was 12% for males and 4% for females [34]. However, not all these deaths are likely to have been related to drug abuse, so the numbers are not directly comparable with those in our study. Besides Finland, Sweden also has a relatively high drug poisoning mortality rate, but a larger proportion of Swedish problem drug users have access to OAT. The estimated coverage of OAT in Finland is 20% among people who use drugs, whereas in Sweden, the proportion is over 70% [25, 35].

This study included all death cases from the three-year period of 2018 to 2020. The beginning of 2020 was strongly influenced by the outbreak of the COVID-19 pandemic causing severe respiratory disease. In Finland, for safety reasons, many health centres, including substance misuse service units offering OAT, reduced their services and supervision of patients. A previous study by our group [36] showed that the monthly findings of BUP, amphetamine and cannabis in post-mortem toxicology increased after the lockdown in March 2020. In England, Aldabergenov et al. [31] found a 64% increase in MET -related deaths in March–June 2020 compared to March–June 2019, with increases in the mortality rate of both in-treatment decedents (22% increase) and decedents not prescribed MET (74% increase). However, there was no increase in BUP-related deaths. We found no increase in the yearly number of deaths among people in or outside of OAT, but the number of individuals was similar in each of the studied three years.

Our study revealed only minor differences between the deceased OAT patients and individuals outside of OAT. Half of the individuals in and outside of OAT died of fatal BUP poisoning. OAT patients had significantly less concomitant use of benzodiazepines, but postmortem findings of both illicit substances and non-prescribed medicines were extremely common in all groups. Concomitant drugs were found regularly in BUP user deaths, e.g. in Australian [11] and US studies [37] in addition to this and the previous Finnish study [12], but the patient characteristics in the other countries differed from our study.

### Strengths and limitations

The high rate of medico-legal cause-of death investigation made it possible to start the study from the premise of post-mortem toxicology data. This study covered all deaths with post-mortem findings of misused BUP with a retrospective review of patient records obtained from substance misuse service units. The response rate to the inquiry to the substance misuse service units was 94%, allowing for population-based assessments. In the absence of national OAT register data, collecting information from all treatment facilities was the only way to obtain the treatment status of these individuals. Due to the challenging and time-consuming data collection, it was not possible to investigate deaths from more than three years, which led to the relatively low number of cases in the groups of OAT patients and those who had been in OAT less than a year before death. Although the sizes of the three study groups were disproportional, given the main goal of the study, we still find these groups justified. Another limitation was that we received patient records only from patients in OAT, not from individuals outside of OAT, so we were unable to reveal differences in their life situations or other factors possibly affecting their deaths.

## Conclusions

In Finland, BUP acts as the substitute for heroin while the smuggled, illicitly sold BUP products are the main cause of concern. Based on the results of our study, Finnish BUP deaths mainly occur outside OAT. As OAT has generally proved to be an effective way to reduce mortality among people with OUD, access to and retention in OAT should be improved. People with OUD should be better informed about unsafe drug use patterns, especially the concomitant use of sedatives. Young individuals dying of fatal drug poisonings seemed to remain outside of OAT. We therefore suggest extending access to OAT to younger age groups and even to those under 18 years.

This study emphasizes the importance of the comprehensive medico-legal cause-of-death investigation as a basis for accurate information when trying to tackle drug-related deaths.
